# Supplementary material for: Addressing the Impact of Surface Roughness on Epsilon-Near-Zero Silicon Carbide Substrates
Source: ACS Photonics. 2023 Aug 22;10(9):3105–14. doi: 10.1021/acsphotonics.3c00476 (PMC10515697; doi:10.1021/acsphotonics.3c00476)
Supplement: Supplementary file 1 — ph3c00476_si_001.pdf [file ph3c00476_si_001.pdf]

Addressing the impact of surface roughness on epsilon-near-zero silicon carbide substrates

SUPPLEMENTARY MATERIAL

David Navajas,[1] José M. Pérez-Escudero,[1] María Elena Martínez-Hernández,[1] Javier Goicoechea, [1] Iñigo Liberal [1,*]
[1]*Department of Electrical, Electronic and Communications Engineering, Institute of Smart Cities (ISC), Public University of Navarre (UPNA), 31006 Pamplona, Spain*

Corresponding author: inigo.liberal@unavarra.es

1. EXPERIMENTAL DATA FOR EACH INDIVIDUAL REFLECTIVITY MEASUREMENT

Due to the intrinsic stochastic nature of surface roughness, a representative response of a rough SiC substrate was estimated by taking four FTIR reflectivity measurements on each sample and subsequently averaging them. Figure S1 shows the experimental data for all individual measurements. The data confirms a small intra-sample variability, though it increases along with the surface roughness RMS, particularly within the SPhP band.

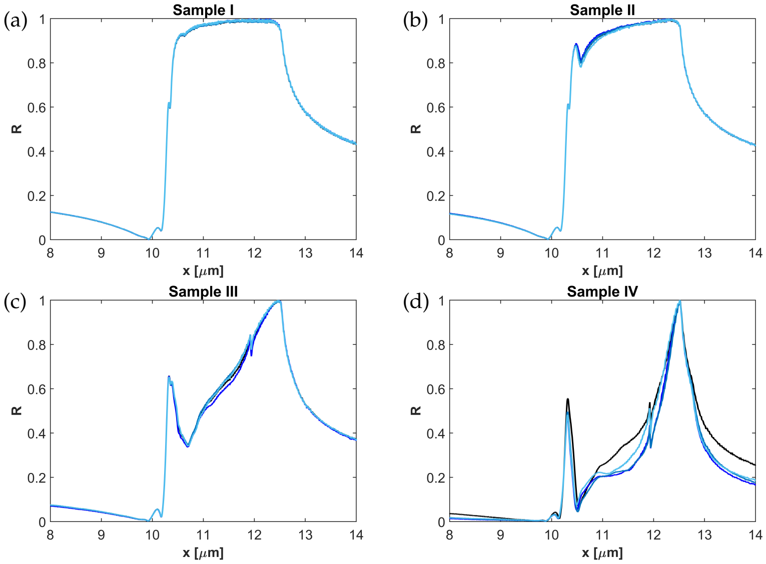

Fig. S1. Experimental data for each individual reflectivity measurement. FTIR reflectivity spectra at four different locations for each sample.

2. RAMAN SPECTROSCOPY MEASUREMENTS

Figure S2 reports a closed-up view of the Raman spectroscopy measurements, as compared to FTIR reflectivity measurements for Sample I and Sample IV. Aside from the strong Raman peaks associated with the transversal ($\lambda = 12.55\,\mu$m, TO) and longitudinal ($\lambda = 10.3\,\mu$m, LO) optical phonons, a small feature can be appreciated at the ZFLO phonon wavelength ($\lambda = 11.93\,\mu$m). However, no individually resolved peaks are found at $\lambda_{II} = 10.35\,\mu m$ and $\lambda_{II} = 10.17\,\mu m$ as observed in the FTIR reflectivity measurements for the sample with a small RMS roughness.

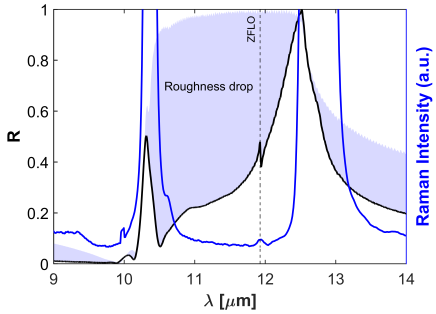

Fig. S2. Close-up view of the Raman spectrum as compared to the reflectivity measurements for Sample I and Sample IV.

3. IMPACT OF THE FTIR RESOLUTION ON THE REFLECTIVITY SPECTRA

Figure S3 presents a comparative analysis of the measured reflectivity spectra for Sample I, for wavenumber resolutions of 0.15 cm^{-1}, 0.3 cm^{-1}, 1 cm^{-1} and 4 cm^{-1}. It can be concluded from the figure that some spectral features can only be recovered operating at a high resolution.

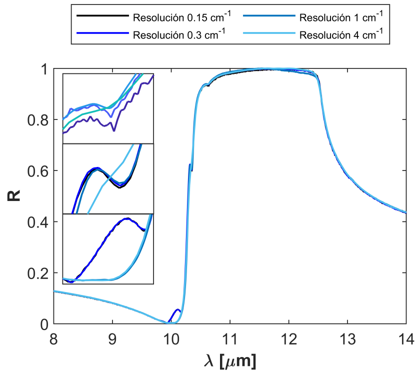

Fig. S3. **Sample I reflectivity spectra as a function of the FTIR wavenumber resolution.**

4. ADDITIONAL NUMERICAL SIMULATIONS RESULTS

A. Data range of the predicted reflectivities

Figure S4 depicts the predicted reflectivity spectra for individual simulations with examples of selected unit-cells P. The data range expanded by all individual simulations is shown as a blue band between the minimum and maximum values. It can be concluded from the figure that individual simulations present resonant peaks that disappear when averaging multiple unit-cell lengths. However, the data range provides a direct estimation of the strength of the individual resonances. The selected examples reported in Fig. 7 of the main text are highlighted with vertical red lines. Figure S5 presents colormaps with all individual numerical simulations employed to calculate the average predicted reflectivity mimicking a rough sample.

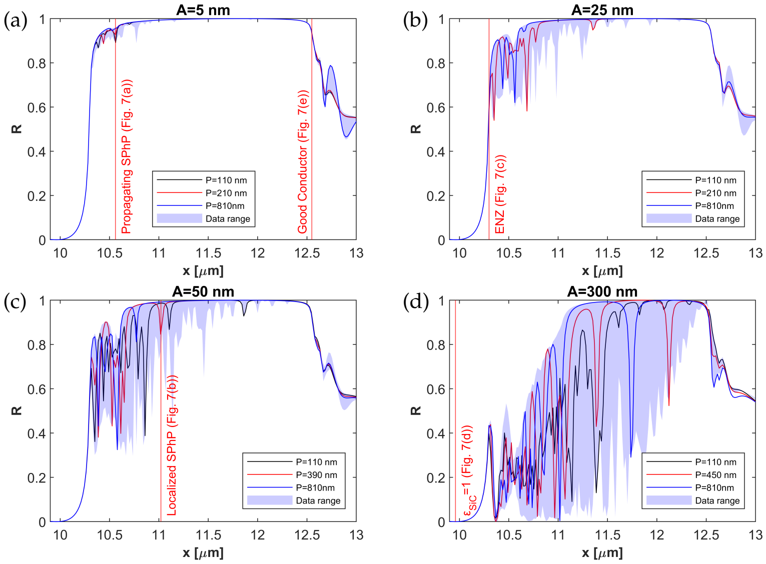

Fig. S4. **Data range for the predicted reflectivities for pitches ranging from 10 nm to 900 nm with a step of 20 nm, and the individual response for selected examples of pitches P.** Each sweep introduces spectral peaks associated with geometrical resonances that disappear when averaging all simulated pitches. Red lines represent the cases shown in Fig. 7 of the main text.

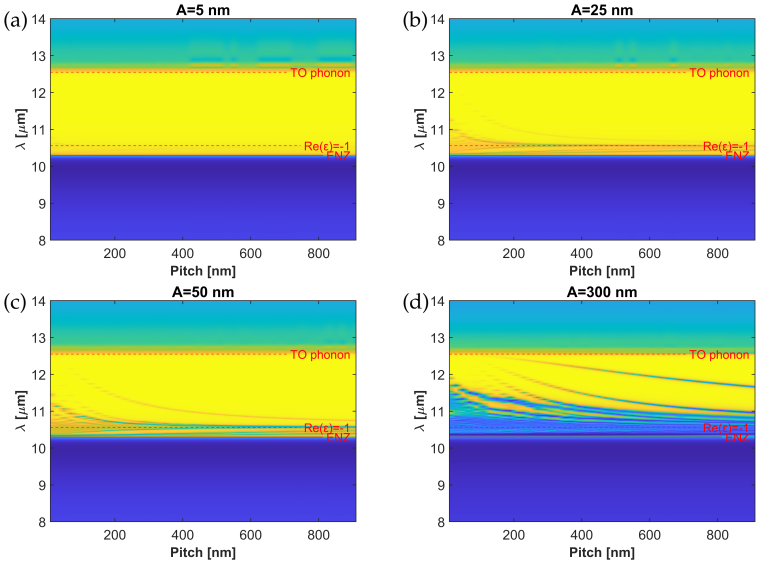

Fig. S5. Predicted reflectivities for pitches ranging from 10 nm to 900 nm with a step of 20 nm. The colormaps include all the numerical simulation data employed in computing the average reflectivity mimicking a rough sample, reported in Fig. 6 of the main text.

B. Oblique incidence reflectivity results

In this supplementary section we carry out additional numerical simulations to investigate the response of rough silicon carbide (SiC) substrates at oblique incidence. First, we calculate the reflectivities of from a flat SiC substrate at 10°, 30°, and 60°angles of incidence, and for parallel polarization, for a better comprehension of the response of rough SiC substrates under oblique incidence (see Figure S6). It can be concluded from the figure that increasing the angle of incidence increases the reflectivity in the ENZ band (roughly between 10 μm and 10.3 μm). On the contrary, the reflectivity in the dielectric bands ($\lambda < 10\mu m$ and $\lambda > 12.55\mu m$) decreases along with the incident angle. Both effects can be ascribed to the change of the transversal impedance as the incident angle increases. These features are expected to be reproduced in the roughness simulations and samples too.

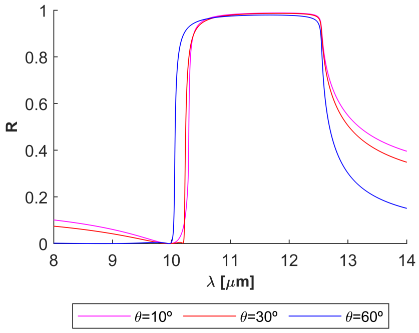

Fig. S6. Calculations for 10°, 30°and 60°with a transverse magnetic (TM) polarization for a flat surface. The most noticeable changes are an increase of the reflectivity in the ENZ band (roughly between 10 μm and 10.3 μm), and a decrease of the reflectivity in the dielectric bands ($\lambda < 10\mu m$ and $\lambda > 12.55\mu m$) as the incident angle increases.

Next, Figure S7 reports numerical simulations for the reflectivity of the rough SiC substrates studied in the main text at oblique incidence. Specifically, we carried out numerical simulations for 10°, 30°, and 60°angles of incidence and parallel polarization. Note that each of these lines

correspond to the averaging after a parameter sweep of the period of the unit-cell as described in the main text. Finally, it can be concluded from Figure S7 that the conclusions drawn in the main text at normal incidence can be extrapolated to a wide range of angles. In fact, major changes in the reflectivity spectra with the angle of incidence relate to those also observed for a flat substrate in Figure S6, while the spectra features associated with ENZ fields and SPhP seems to be stable with changes of the angle of incidence.

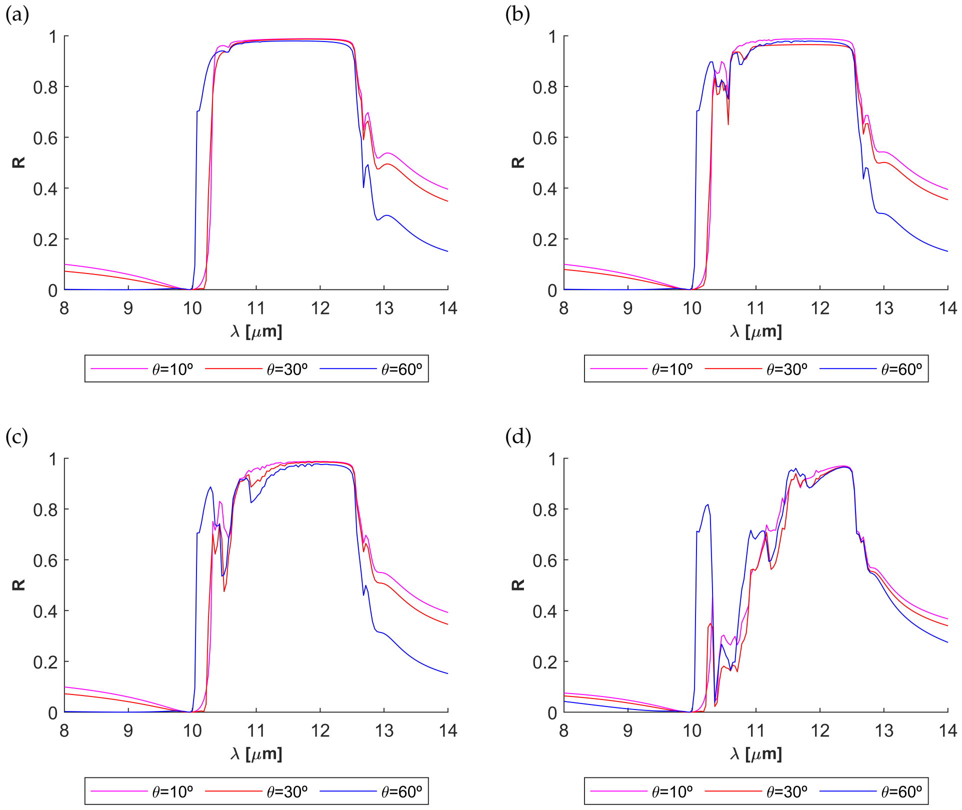

Fig. S7. Predicted oblique reflectivities for pitches ranging from 10 nm to 900 nm with a step of 20 nm at angles of 10°, 30°and 60°. The numerical simulations show that the angular dependence of rough SiC substrates essentially follows that of a flat substrate. Therefore, the conclusions drawn in the main text at normal incidence can be extrapolated to a wide range of incident angles.
